# Radar Tracking with an Interacting Multiple Model and Probabilistic Data Association Filter for Civil Aviation Applications

**DOI:** 10.3390/s130506636

**Published:** 2013-05-17

**Authors:** Shau-Shiun Jan, Yu-Chun Kao

**Affiliations:** Institute of Civil Aviation, National Cheng Kung University, Tainan 70101, Taiwan; E-Mail: kaocnsl@gmail.com

**Keywords:** radar, Kalman filter, interacting multiple model, probabilistic data association filter, air traffic management

## Abstract

The current trend of the civil aviation technology is to modernize the legacy air traffic control (ATC) system that is mainly supported by many ground based navigation aids to be the new air traffic management (ATM) system that is enabled by global positioning system (GPS) technology. Due to the low receiving power of GPS signal, it is a major concern to aviation authorities that the operation of the ATM system might experience service interruption when the GPS signal is jammed by either intentional or unintentional radio-frequency interference. To maintain the normal operation of the ATM system during the period of GPS outage, the use of the current radar system is proposed in this paper. However, the tracking performance of the current radar system could not meet the required performance of the ATM system, and an enhanced tracking algorithm, the interacting multiple model and probabilistic data association filter (IMMPDAF), is therefore developed to support the navigation and surveillance services of the ATM system. The conventional radar tracking algorithm, the nearest neighbor Kalman filter (NNKF), is used as the baseline to evaluate the proposed radar tracking algorithm, and the real flight data is used to validate the IMMPDAF algorithm. As shown in the results, the proposed IMMPDAF algorithm could enhance the tracking performance of the current aviation radar system and meets the required performance of the new ATM system. Thus, the current radar system with the IMMPDAF algorithm could be used as an alternative system to continue aviation navigation and surveillance services of the ATM system during GPS outage periods.

## 1. Introduction

According to the International Civil Aviation Organization's plan, the current technology trend is to develop and implement Communications, Navigation, Surveillance, and Air Traffic Management (CNS/ATM) systems based on the Global Positioning System (GPS) to replace legacy Air Traffic Control (ATC) systems based on ground-based radar [1]. The Next Generation Air Transportation System (NextGen) will modernize the air traffic control system in the United States, and many of the foundational elements required to meet the predicted capacity and efficiency improvements rely on widespread use of precision Positioning, Navigation, and Timing (PNT) services provided by the GPS [2]. The Federal Aviation Administration (FAA) needs to ensure a sufficient backup PNT capability to reduce the risks of aviation users if GPS becomes unavailable. As described in the FAA Flight Plan [2], the air traffic system of the future will be much more dependent on GPS. This strategic goal will not be achievable without a NextGen Alternate PNT (APNT), especially in the event of a GPS loss of service. The APNT program ensures alternate PNT services provided by the ATC system, minimizes the economic impact of GPS outages, and supports air transportation's timing needs. The existing legacy navigation and surveillance infrastructure, *i.e.*, VHF Omnidirectional radio Range (VOR), Distance Measuring Equipment (DME), Non-Directional Beacon (NDB), and secondary radar, may not achieve the desired level of performance for NextGen operation [3].

To improve ATC tracking performance, one solution is to upgrade the ATC radar beacon system. However, this solution is expensive. A more cost-effective solution is to improve the ATC tracking algorithms, which does not require the implementation of new radar facilities. The present study thus proposes a radar tracking algorithm that meets the requirements of APNT systems. The major challenge and difficulty for ATC tracking is tracking target maneuvering in a cluttered environment [4–7]. Bar-Shalom and Blom [4] introduce a tracking algorithm called the Interacting Multiple Model (IMM) estimator, which provides tracking estimates with significant noise reduction and fast response to sequences of aircraft maneuver modes [4,5]. However, the tracking of an aircraft in a cluttered environment might be a challenge due to the several observations for a single aircraft under such environment [6,7]. That is, some tracking measurements do not originate from the target aircraft. Therefore, the present study utilizes the Probabilistic Data Association (PDA) filter [6,7] to assign weights to the validated measurements. The PDA filter can extend the tracking capability to a highly cluttered environment. In order to gain possible improvement on the tracking performance, this study combines the IMM estimator and PDA filters to create an IMMPDA filter (IMMPDAF). Most studies on radar tracking algorithms use simulated data for analysis. To further evaluate the IMMPDAF, this study uses real flight radar data collected by the Civil Aeronautics Administration (CAA) of Taiwan. A filter that uses the nearest neighbor method and the standard Kalman filter (NNKF) is commonly used for radar tracking systems. Therefore, this paper compares the tracking performance of the IMMPDAF and the NNKF in terms of positioning accuracy. Finally, the validation of the IMMPDAF under APNT requirements is discussed. Due to the complexity of the IMMPDAF, this paper also conducts a computational load study on the IMMPDAF and the NNKF.

The rest of this paper is organized as follows: Section 2 introduces the algorithms of the IMM estimator and the PDA filter. Then, the IMM estimator and the PDA filter are integrated. The dynamic model setting of the IMMPDAF for the ATC scenario is also discussed in Section 2. The implementation of the IMMPDAF to a radar system is discussed in Section 3. The tracking performance of the IMMPDAF is compared with that of the NNKF. In addition, the computation loads of these filters are evaluated. Section 4 concludes this work.

## 2. IMMPDAF

### 2.1. IMM Estimator

The IMM estimator is a good candidate for a radar tracking system [4]. The IMM estimator provides significant noise reduction and a fast response. Previous investigations have found that the IMM estimator is a cost-effective technique for tracking a maneuvering target [4]. In the IMM estimator, several Kalman filters are used in parallel. Each Kalman filter uses a different dynamic model. The IMM estimator can be separated into four steps. In the first step, the state estimate and covariance are mixed. These mixed estimates and covariances are the inputs of the Kalman filter. The equations are:

(1) $$U_{ij}\;(k-1)=\frac{p_{ij}u_{i}\;(k-1)}{\overset{r}{\underset{l=1}{\text{∑}}}\;p_{lj}u_{l}\;(k-1)}$$

(2) $$X_{j}^{0}\;(k-1)=\overset{r}{\underset{i=1}{\text{∑}}}\;X_{i}^{+}(k-1)\;U_{ij}\;(k-1)$$

(3) $$P_{j}^{0}\;(k-1)=\overset{r}{\underset{i=1}{\text{∑}}}\begin{array}{c} U_{ij}\;(k-1)\;\{P_{i}^{+}\;(k-1)+\left[X_{i}^{+}\;(k-1)-X_{j}^{0}\;(k-1)\right]\;{\left[X_{i}^{+}\;(k-1)-X_{j}^{0}\;(k-1)\right]}^{T}\} \end{array}$$

In Equations (1–3), the subscripts *i*, *j*, and *l* indicate different Kalman filters. There are up to *r* different dynamic models of Kalman filter, the total number for *u_i_*, $X_{j}^{0}$, $X_{i}^{+}$, $P_{j}^{0}$ and $P_{i}^{+}$ is *r*, and the total number for *U_ij_* and *p_ij_* is *r*^2^. *u_i_* (*k* − 1) represents the model probabilities in the previous time (*k* − 1). Since the initial model probabilities have little impacts on the results over time, they can be conveniently chosen. In this study, the initial model probabilities are set to be rectangular functions. *p_ij_* is an element of the Markov transition matrix; there is probability *p_ij_* that the target will transit from model *i* to model *j* at each time step. *U_ij_* represents the conditional probability of the target in the *j* model state, which transited from the *i* model state. According to [5], the IMM algorithm performance is robust to the choice of transition matrix. $X_{i}^{+}(k-1)$ and $P_{i}^{+}(k-1)$ are the updated state and covariance, respectively, from the Kalman filter in the previous time step (*k* − 1). $X_{j}^{0}\;(k-1)$ and $P_{j}^{0}\;(k-1)$ are the mixed state and the mixed covariance, respectively, for each Kalman filter as inputs at time *k*. Then, the second step implements the multiple models of the Kalman filter:

(4) $$\{\begin{array}{c} X_{j}^{-}\;(k)=F_{j}\cdot X_{j}^{0}\;(k-1)\\ P_{j}^{-}\;(k)=F_{j}\cdot P_{j}^{0}\;(k-1)\cdot F_{j}^{T}+Q_{j} \end{array}$$

(5) $$\{\begin{array}{c} K_{j}=P_{j}^{-}\;(k)\cdot H_{j}^{T}{\;(H_{j}\cdot P_{j}^{-}\;(k)\cdot H_{j}^{T}+R_{j})}^{-1}\\ X_{j}^{+}\;(k)=X_{j}^{-}\;(k)+K_{j}\cdot \left(Z\;(k)-H_{j}\cdot X_{j}^{-}\;(k)\right)\\ P_{j}^{+}\;(k)=(I-K_{j}\cdot H_{j})\cdot P_{j}^{-}\;(k) \end{array}$$

The details of Kalman filter can be found in [8]. In Equations (4,5) the subscript *j* indicates it is the *j* th Kalman filter, and Equations (4,5) are applied for each Kalman filter. In Equation (4)*F_j_* is the dynamic model and *Q_j_* is the covariance of the process noise. $X_{j}^{-}\;(k)$ and $P_{j}^{-}\;(k)$ are the uncorrected predicted state and covariance, respectively. In Equation (5)*H_j_* is the observation matrix, *R_j_* is the covariance of the measurement noise, and *K_j_* is the Kalman gain. *Z*(*k*) is the measurement at time *k* and *I* is the identity matrix. $X_{i}^{+}(k)$ and $P_{i}^{+}(k)$ are the corrected predicted state and covariance, respectively. In the third step, the updated model probabilities are calculated as:

(6) $$\{\begin{array}{c} S_{j}\;(k)=H_{j}\cdot P_{j}^{-}\;(k)\cdot H_{j}^{T}+R_{j}\\ {\tilde{y}}_{j}\;(k)=Z\;(k)-H_{j}\cdot X_{j}^{-}\;(k)\\ d_{j}^{2}\;(k)={\tilde{y}}_{j}{\;(k)}^{T}S_{j}{\;(k)}^{-1}{\tilde{y}}_{j}\;(k)\\ \Lambda_{j}\;(k)=\frac{\exp\left[\frac{-d_{j}^{2}\;(k)}{2}\right]}{\sqrt{\;|\;2\pi \times S_{j}\;(k)\;|}} \end{array}$$

(7) $$u_{j}\;(k)=\frac{u_{j}\;(k-1)\cdot \Lambda_{j}\;(k)}{\overset{r}{\underset{i=1}{\text{∑}}}u_{j}\;(k-1)\cdot \Lambda_{i}\;(k)}$$

In Equation (6), the subscript *j* indicates it is the *j*th Kalman filter; exp[] means the exponential operation and || represents calculating the determinant of the matrix. The likelihood function Λ*_j_* is the probability density function of the estimated measurement *H_j_* · $X_{j}^{-}\;(k)$, which is a normal distribution with mean *Z*(*k*) and covariance *S_j_*(*k*). In Equation (7), the subscripts *i* and *j* indicate different Kalman filters. *u_j_*(*k*) represents the latest model probabilities at time *k*. In the last step, the corrected predicted state $X_{j}^{+}\;(k)$ and $P_{j}^{+}\;(k)$ covariance from the Kalman filters are combined. The weighting factor is the latest model probabilities *u_j_*(*k*):

(8) $$X\;(k)=\overset{r}{\underset{j=1}{\text{∑}}}X_{j}^{+}\;(k)\cdot u_{j}\;(k)$$

(9) $$P\;(k)=\overset{r}{\underset{j=1}{\text{∑}}}\;u_{j}\;(k)\;\left\{\;P_{j}^{+}\;(k)+\left[X_{j}^{+}\;(k)-X\;(k)\right]\;{\left[X_{j}^{+}\;(k)-X\;(k)\right]}^{T}\right\}$$

*X*(*k*) and *P*(*k*) are the combinations of the system state and covariance from 1 to *r*th Kalman filters, respectively. Using Equations (1–9), the IMM estimator calculates the system state and covariance from time *k* − 1 to *k*.

### 2.2. PDA Filter

The PDA filter is designed for tracking a target in a cluttered environment based on the Kalman filter [6,7]. The PDA filter obtains an estimator which incorporates all the returning measurements that might originate from the target of interest rather than select only one of them. The resulting estimator sets a gate to determine whether the measurements are valid. The association probabilities of the target being tracked for each validated measurement are then calculated. These probabilities are assigned to all of the validated measurements with different weights. Next, the validated measurements are combined with different weights based on location, and then the combined measurement is used to update the state estimate of the target. The PDA filter can extend the tracking capability to a highly cluttered environment.

The probabilities are used for weighting the correction and covariance in the PDA filter. The basic assumptions and theories for the PDA filter can be found in [6,7]. Here, a brief introduction of its functions and equations is given. Because the PDA filter is based on the Kalman filter, the first equations are the same as those for the Kalman filter:

(10) $$\{\begin{array}{c} X_{j}^{-}\;(k)=F_{j}\cdot X_{j}^{+}\;(k-1)\\ {\hat{z}}_{j}\;(k)=H_{j}\cdot X_{j}^{-}\;(k)\\ P_{j}^{-}\;(k)=F_{j}\cdot P_{j}^{+}\;(k-1)\cdot F_{j}^{T}+Q_{j} \end{array}$$

Similarly, the first step is to predict the system state and covariance. In Equation (10), the subscript *j* indicates it is the *j*th PDA filter, $X_{j}^{+}\;(k-1)$ is the calculated state at time *k* − 1, *F* is the dynamic model, and $X_{j}^{-}\;(k)$ is the predicted state at time *k. H* is the observation matrix and *zˆ_j_*(*k*) is the predicted measurement at time *k*. $P_{j}^{+}\;(k-1)$ is the calculated covariance at time *k* − 1 and *Q_j_* is the process noise covariance. The second step is to determine which measurements can be used to update the state and covariance:

(11) $$\{\begin{array}{c} Z_{i}^{j}\;(k)=z_{i}\;(k)-{\hat{z}}_{j}\;(k)\\ S_{j}\;(k)=H_{j}\cdot P_{j}^{-}\;(k)\cdot H_{j}^{T}+R_{j} \end{array}$$

(12) $$Z_{i}^{j}{\;(k)}^{T}\cdot S_{j}\;(k)\cdot Z_{i}^{j}\;(k)\leq \gamma$$

The main purpose of Equation (11) and (12) is to validate the measurements by Chi-Square test, and it includes all the measurements returned by all the radars. In Equation (11), the total number of measurements is unknown; the subscript *i* indicates it is the *i*th measurement. $Z_{i}^{j}$ is the innovation of *i*th measurement for *j*th PDA filter, *z_i_*(*k*) is the *i*th measurement at time *k* and *R* is the covariance of the measurement noise. Equation (12) is the validation equation. *γ* is the threshold corresponding to the gate probability. *γ* can be obtained from Chi-Square tables for a chosen gate probability. Once the *i*th measurement passes the Chi-Square test in Equation (12), it can be utilized in the rest of the PDA filter. Then, the association probabilities of each validated measurement are calculated as:

(13) $$\{\begin{array}{c} V_{j}\;(k)=(4\pi /3)\cdot \gamma^{3/2}\cdot \sqrt{\;|\;S_{j}\;(k)\;|}\\ L_{i}^{j}\;(k)=\frac{N\;[z_{i}\;(k);{\hat{z}}_{j}\;(k);S_{j}\;(k)]\cdot P_{D}}{m/V_{j}\;(k)} \end{array}$$

(14) $$\beta_{i}^{j}\;(k)=\{\begin{array}{c} \frac{L_{i}^{j}\;(k)}{1-P_{D}\;\cdot \;P_{G}+\sum_{n=1}^{m}\;L_{n}^{j}\;(k)},i=1,\cdots m\\ \frac{1-P_{D}\;\cdot \;P_{G}}{1-P_{D}\;\cdot \;P_{G}+\sum_{n=1}^{m}\;L_{n}^{j}\;(k)},i=0 \end{array}$$

In Equation (13), the subscript *j* indicates it is the *j*th PDA filter, *V*(*k*) is the volume of the validation region, *m* is number of total validation measurements, and *N*[*z_i_*(*k*);*zˆ*(*k*);*S*(*k*)] represents the normal distribution, centered at *zˆ*(*k*)with variance *S*(*k*). $L_{i}^{j}$ is the likelihood of the *i*th measurement for *j*th PDA filter, $\beta_{i}^{j}$ is the associated probability of the *i*th measurement for *j*th PDA filter. In Equations (13) and (14), *P_D_* and *P_G_* are the target detection probability and the gate probability, respectively. *β_i_*(*k*) is the association probability of the validated measurements. Note that *β*_0_(*k*) is the probability that all measurements are not valid [7]. The suggested values of *P_D_* and *P_G_* can be found in [5]. *P_D_* is required to be larger than 90% for primary surveillance radar sensor and 98% for secondary surveillance radar sensor. *P_G_* is the probability that the true measurement from the target is detected and validated; and *P_G_* is usually chosen to be at least 95%. If *P_G_* is set too low, the gate *γ* will be too small that no measurement can pass the validation and it could be end up losing track. If *P_G_* is set too high on the other hand, the gate *γ* will be too large that causes too much false alarms (*i.e.*, not true measurement from the target) and reduction on the accuracy [5]. The last step is to update the system state and covariance. In this step, only the validated measurements and their association probabilities are used:

(15) $$\{\begin{array}{c} v_{j}\;(k)=\sum_{i=1}^{m}\;\beta_{i}^{j}\cdot Z_{i}^{j}\;(k)\\ W_{j}\;(k)=P_{j}^{-}\;(k)\cdot H_{j}^{T}\cdot S_{j}{\;(k)}^{-1}\\ P_{j}^{\prime \prime }\;(k)=P_{j}^{-}\;(k)-W_{j}\;(k)\cdot S_{j}\;(k)\cdot W_{j}{\;(k)}^{T}\\ P_{j}^{\prime \prime \prime }\;(k)=W_{j}\;(k)\cdot \left[\overset{m}{\underset{i=1}{\text{∑}}}\;\beta_{i}^{j}\cdot Z_{i}^{j}\;(k)\cdot Z_{i}^{j}{\;(k)}^{T}-v_{j}\;(k)\cdot v_{j}{\;(k)}^{T}\right]\cdot W_{j}{\;(k)}^{T} \end{array}$$

(16) $$\{\begin{array}{c} X_{j}^{+}\;(k)=X_{j}^{-}\;(k)+W_{j}\;(k)\cdot v_{j}\;(k)\\ P_{j}^{+}\;(k)=\beta_{0}^{j}\cdot P_{j}^{-}1-\beta_{0}^{j}+(1-\beta_{0}^{j})\cdot P_{j}^{\prime \prime }\;(k)+P_{j}^{\prime \prime \prime }\;(k) \end{array}$$

In Equations (15,16), the subscript *j* indicates it is the *j*th PDA filter, *v*(*k*) is combined innovation; *W*(*k*) is Kalman gain; *P*″(*k*) and *P*‴ (*k*) are the covariances of state updated. In Equation (16)*X*^+^(*k*) and *P*^+^(*k*) are the calculated system state and covariance, respectively at time *k*. The propagation of the PDA filter is shown in Equations (10–16).

### 2.3. IMMPDA Filter

**Figure 1.**
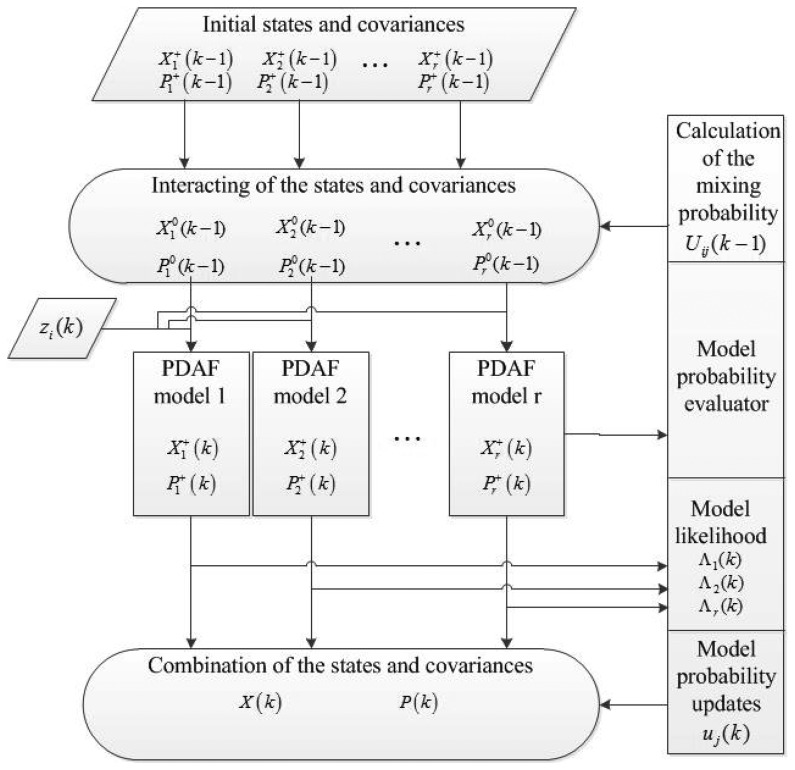
Flow chart of the IMMPDAF with three models.

The IMM estimator and PDA filter can be combined as shown in Figure 1. The Kalman filters in the IMM estimator are replaced by the PDA filters. At the top, Equations (1–3) are implemented to deal with the data from the previous epoch. Then, the PDA filters [Equations (10–16)] use the data from Equations (1–3) as the initial values and generate the calculated states and covariances. The next step is to use Equations (6,7) to obtain the latest model probabilities. The last step is to utilize Equations (8,9) to combine the states and covariances with different weights, which are related to the latest model probabilities. The combinations of states and covariances, *X*(*k*) and *P*(*k*) are the outputs of the IMMPDAF. In Figure 1, the IMMPDAF has three different models of PDA filter. The number of models depends on the number of dynamic motions of the target. In this paper, the tracking target is a commercial airline aircraft. The selection of suitable motion models for an ATC system is discussed in the next section.

### 2.4. Tuning of IMMPDAF

As described above, the IMMPDAF can be considered as a modification of the Kalman filter. The Kalman filter is tuned by changing the process noise covariance *Q* matrices and the measurement noise covariance *R* matrices. The matrices *Q* and *R* adjust the Kalman gain. The *R* matrices represent the accuracy of the measurement device; larger *R* matrices mean that the measurements can be trusted less. In most practical situations, matrices *Q* and *R* are unknowns. In this paper, a run-time estimate method [9] is used to obtain the measurement noise covariance *R*. The adaptive measurement noise covariance is estimated in Equation (17). *v*(*n*)is the innovation in time epoch *n*, obtained from Equation (15); and *v^avg^*(*k*) is average innovation value from time 1 to time *k*:

(17) $$\{\begin{array}{c} v_{j}^{\text{avg}}\;(k)=\frac{\overset{k}{\underset{n=1}{\text{∑}}}\;v_{j}\;(n)}{k}\\ R_{j}\;(k)=\frac{\overset{k}{\underset{n=1}{\text{∑}}}\left[v_{j}\;(n)-v_{j}^{\text{avg}}\;(k)\right]\;{\left[v_{j}\;(n)-v_{j}^{\text{avg}}(k)\right]}^{T}}{k} \end{array}$$

From the conclusions of [9], the run-time estimate method can handle changes of measurement noise covariance, and converge faster than that of a standard Kalman filter.

### 2.5. Dynamic Models of Commercial Airline Aircraft

The key to successful target tracking is selecting suitable dynamic models that fit the target motion [10,11]. Ideally, each motion should have a correct dynamic model. In an ATC scenario, the motions of commercial airline aircraft can be summarized as ascent, descent, en-route, turning, and change of speed. These motions can be described as three basic dynamics: constant velocity motion, accelerated motion, and turning motion [11,12]. Following [11–15] and our previous work [16], this study uses the constant velocity model, the acceleration model, and the turning rate estimator model, respectively given in Equations (18–20), respectively, for the IMMPDAF. For ATC radar tracking, the measurement is the target 3-dimensional position (*x*, *y*, *z*) of the tracking target in WGS-84 coordinates. In Equations (18–20), *X* is the system state, *x*, *y*, *z* are the positions, *ẋ, ẏ, ż* are the velocities, and *ω* is the turning rate. *F* represents the dynamic model and *t* is the time interval. Generally speaking, the time epoch is close to 5 seconds. *Q* is the covariance of process noise and *σ* is zero-mean Gaussian white noise. The *σ* value is tuned by experience and testing, and the value is related to the measurement noise covariance:

(18) $$\{\begin{array}{c} X=\left[\begin{array}{c} x\\ y\\ z\\ \dot{x}\\ \dot{y}\\ \dot{z} \end{array}\right]\;,F=\left[\begin{array}{cc} I_{3} & tI_{3}\\ 0 & I_{3} \end{array}\right]\;,\\ Q=\left[\begin{array}{cc} \frac{t^{2}}{2}I_{3} & 0\\ 0 & tI_{3} \end{array}\right]\;\sigma \end{array}$$

(19) $$\{\begin{array}{c} X=\left[\begin{array}{c} x\\ y\\ z\\ \dot{x}\\ \dot{y}\\ \dot{z} \end{array}\right]\;,F=\left[\begin{array}{cc} I_{3} & tI_{3}\\ 0 & I_{3} \end{array}\right]\;,\\ Q=\left[\begin{array}{cc} \frac{t^{3}}{3}I_{3} & \frac{t^{2}}{2}I_{3}\\ \frac{t^{2}}{2}I_{3} & tI_{3} \end{array}\right]\;\sigma \end{array}$$

(20) $$\{\begin{array}{c} X=\left[\begin{array}{c} x\\ y\\ z\\ \dot{x}\\ \dot{y}\\ \dot{z}\\ \omega \end{array}\right],F=\left[\begin{array}{ccccccc} 1 & 0 & 0 & t & \frac{-\omega t^{2}}{2} & 0 & 0\\ 0 & 1 & 0 & \frac{\omega t^{2}}{2} & t & 0 & 0\\ 0 & 0 & 1 & 0 & 0 & t & 0\\ 0 & 0 & 0 & \left(1-\frac{\omega^{2}t^{2}}{2}\right) & -\omega t & 0 & 0\\ 0 & 0 & 0 & \omega t & \left(1-\frac{\omega^{2}t^{2}}{2}\right) & 0 & 0\\ 0 & 0 & 0 & 0 & 0 & 1 & 0\\ 0 & 0 & 0 & 0 & 0 & 0 & 1 \end{array}\right],\\ Q=\left[\begin{array}{ccccccc} \frac{t^{4}}{4} & 0 & 0 & \frac{t^{3}}{2} & 0 & 0 & 0\\ 0 & \frac{t^{4}}{4} & 0 & 0 & \frac{t^{3}}{2} & 0 & 0\\ 0 & 0 & \frac{t^{4}}{4} & 0 & 0 & \frac{t^{3}}{2} & 0\\ \frac{t^{3}}{2} & 0 & 0 & t^{2} & 0 & 0 & 0\\ 0 & \frac{t^{3}}{2} & 0 & 0 & t^{2} & 0 & 0\\ 0 & 0 & \frac{t^{3}}{2} & 0 & 0 & t^{2} & 0\\ 0 & 0 & 0 & 0 & 0 & 0 & \frac{t^{2}\sigma_{\omega }}{\sigma } \end{array}\right]\sigma \end{array}$$

## 3. Experimental Results and Discussion

The experiment data are from the CAA of Taiwan. The data formats include radar data (ASTERIX AC048) and ADS-B (ASTERIX AC021). The radar data were recorded from local time 12:00:00 midnight on 28 March 2012. Hundreds of commercial airline aircraft were tracked and recorded. Two cases are used here to investigate whether the performance of the IMMPDAF significantly varies in the area surrounding Taiwan. The first case target was from east head to Taiwan and the second case target flight away from Taiwan to north. The radar data was processed using the IMMPDAF and the NNKF. The ADS-B data have the target ‘Callsign’ and GPS calculated positions; these were set as the initial values of the two methods and regarded as the true positions. As described in the previous section IMMPDAF includes three filters of different dynamic models shown in Equations (18–20). The parameters of initial model probabilities and transition matrix are shown in Equation (21). The initial values of model probabilities have minor effect on the performance, and the IMMPDAF is very robust to the choice of transition matrix. Since the values set for *P_D_* is 0.9 and *P_G_* is 0.999, and the degree of freedom is 3, the gate threshold obtained from Chi-square table is approximately 16.

(21) $$\{\begin{array}{c} u_{\text{initial}}=\left[\begin{array}{ccc} 1/3 & 1/3 & 1/3 \end{array}\right]\\ p=\left[\begin{array}{ccc} 0.8 & 0.1 & 0.1\\ 0.1 & 0.8 & 0.1\\ 0.1 & 0.1 & 0.8 \end{array}\right] \end{array}$$

The performance requirements for APNT can be found in [17]. The minimum performance requirements for APNT are shown in Table 1. The FAA categorized the airspace into several zones [17]. En-route is the airspace at flight level (FL) 180 to FL 600 (18,000 to 60,000 feet). Terminal is the airspace starting at 500 feet above the ground and extending out to 5 statute miles from the airport; it then goes up at a 2-degree angle to FL 180. Under 500 feet, the APNT system needs to support the LNAV/non-precision approach. Accuracy (95%) is defined as the errors in the horizontal 95% under the accuracy requirement. That is, according to a normal distribution, 95% of error (error mean + 2 standard deviations) has to be under the accuracy requirement.

### 3.1. Case 1

**Figure 2.**
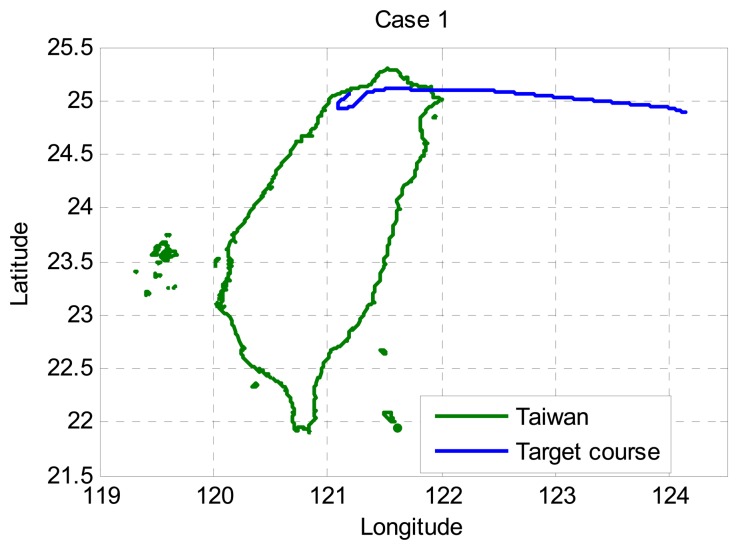
Flight path for case 1 (target heading toward Taiwan).

**Figure 3.**
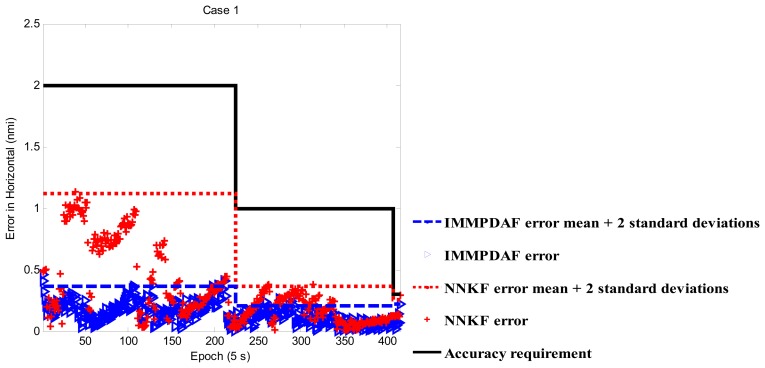
Performance of IMMPDAF and NNKF for Case 1.

**Figure 4.**
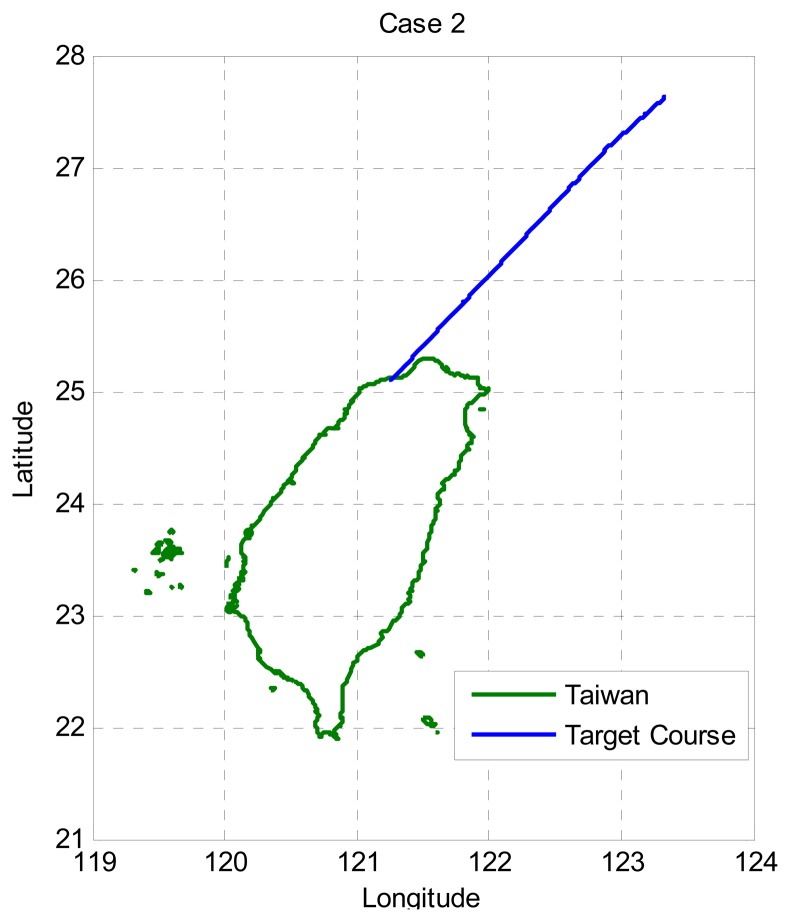
Flight path for Case 2 (target flying away from Taiwan).

**Figure 5.**
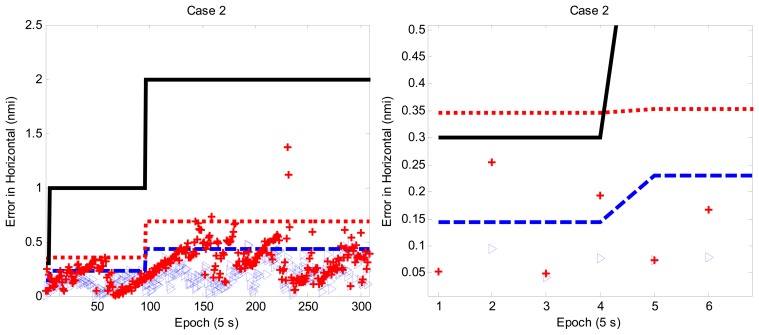
Performance of IMMPDAF and NNKF for Case 2.

In Figure 2, the target heads to Taiwan Taoyuan International Airport (IATA: TPE, ICAO: RCTP) from east to west. The interval between epochs is 5 s. Note that a lot of surveillance radar systems have a scan time of close to 5 s. For example, ASR-12 surveillance radar has a scan time of 4 to 6 seconds. The tracking performance is shown in Figure 3. In case 1, the target starting at en-route airspace, so the accuracy requirement begins from 2 nmi. As shown in Figure 3, the NNKF experiences the instability at the beginning and the IMMPDAF performs more stable. The reason of the difference is the strategy to deal with the low quality measurements. While the NNKF just chooses to trust one of the measurements and updates, the IMMPDAF gives all the validated measurements weightings and updates with all of them. From Figure 3, the tracking performance of both the IMMPDAF and the NNKF meets the accuracy requirement. The IMMPDAF outperforms the NNKF, as shown in Table 2. The legends for Figures 3, 4 and 5 are illustrated below.

### 3.2. Case 2

In Case 2, the target takes off from Taiwan Taoyuan International Airport and flies northeast as depicted in Figure 4. The tracking performance is shown in the upper plot of Figure 5, and the bottom plot of Figure 5 is the zoom-in plot of the upper one which shows the tracking performance for the LNAV/non-precision approach region. Note that the NNKF results initially exceed the accuracy requirement of APNT whereas the IMMPDAF results remain under the accuracy requirement. That is, the NNKF method does not meet the accuracy requirement (95%) in this case. As a result, the IMMPDAF outperforms the NNKF.

The mean accuracies of the horizontal positioning are summarized in Table 2. The IMMPDAF outperforms the NNKF for all tested cases. In comparison to the use of the NNKF, the average improvement on the positioning (tracking) performance gained from the proposed IMMPDAF is about 50%. The overall mean accuracies of the horizontal positioning are also listed in Table 2.

### 3.3. Computation Load

For all the experiments, the computations were performed in MATLAB (R2008a). A PC with an Intel Core 2 Duo E7500 2.93-GHz CPU and 2 GB of RAM was used. The computation performance was measured using a MATLAB function. The computation performance results are listed in Table 3. The values are total seconds that running a case needed. Each test runs 10 times and to obtain the average result. The average result is shown that both algorithms in the single target ATC radar tracking scenario are below the radar scan time an epoch (5 seconds).Without coding optimization, the IMMPDAF requires about nine times than that of the NNKF.

## 4. Conclusions

This paper presented a filter based on the Interacting Multiple Model (IMM) estimator and the Probabilistic Data Association (PDA) filter, and this filter is thus called IMMPDAF, which was applied to the radar tracking system. The real flight radar data was used to evaluate the tracking performance of the IMMPDAF, and the conventional Nearest Neighbor Kalman Filter (NNKF) was used as the baseline to show the performance gained from the proposed filter. Experimental results show that the tracking performance of the IMMPDAF is better than that of the NNKF. In one test case (*i.e.*, Case 2), the IMMPDAF meets the accuracy requirement of the Alternate Positioning, Navigation, and Timing (APNT), but the NNKF results exceeded the APNT accuracy requirement for some periods of time. Although the computation load of the IMMPDAF is nine times that of the NNKF, the IMMPDAF is still suitable for ATC radar since processing time of each epoch is below the radar scan time (5 s). The proposed IMMPDAF gained about 50% improvement on tacking performance than that of the NNKF. Importantly, the radar tracking with the proposed IMMPDAF met the performance requirements of the APNT. That is, the current radar with the proposed IMMPDAF is capable to provide the navigation and surveillance services for the Air Traffic Management (ATM) system during periods of GPS outages.

## Figures and Tables

**Table 1.** Required performance for APNT.

	Navigation	Surveillance	
	Accuracy (95%)	Separation	*NAC_p_*
En-route	10 nmi	5 nmi	0.1 nmi
	4 nmi		
	2 nmi		
Terminal	1 nmi	3 nmi	0.05 nmi
LNAV	0.3 nmi		

**Table 2.** Overall mean accuracies of the horizontal positioning for IMMPDAF and NNKF.

**Accuracy (95%)**	**IMMPDAF**	**NNKF**
**Cases 1**	0.311 nmi	0.921 nmi
**cases 2**	0.397 nmi	0.582 nmi
**AVERAGE**	0.354 nmi	0.751 nmi

**Table 3.** Computation performance of IMMPDAF and NNKF for Cases 1 to 2. Lower values are better.

**Cost Time (s)**	**IMMPDAF**	**NNKF**
**CASE 1**	19.754 s	2.249 s
**CASE 2**	25.110 s	2.734 s
**AVERAGE**	22.432 s	2.491 s
